# Focal epilepsy presenting as tongue tremor: A case report

**DOI:** 10.1002/ccr3.5478

**Published:** 2022-02-23

**Authors:** Mehri Salari, Kamran Rezaei, Alimohammad Mirdehghan, Arya Behzadi, Masoud Etemadifar

**Affiliations:** ^1^ Functional Neurosurgery Research Center Shohada Tajrish Comprehensive Neurosurgical Center of Excellence Shahid Beheshti University of Medical Sciences Tehran Iran; ^2^ Student Research Committee School of Medicine Shahid Beheshti University of Medical Science Tehran Iran; ^3^ Department of Functional Neurosurgery Medical School Isfahan University of Medical Science Isfahan Iran

**Keywords:** dyskinesia, focal epilepsy, tongue, tremor

## Abstract

Plenty of etiologies are reported to cause tongue tremor. Focal epilepsy presenting as isolated tongue tremor is a rare condition, suggesting how variable the focal seizure presentation may be. This paper reports a case of focal epilepsy due to presence of a cavernous angioma in the region of cortical motor area related to tongue movements. It is an clinical example of pathological conditions affecting the tongue area in motor homunculus.

## Graphical abstract

Focal epilepsy presenting as isolated tongue tremor is a rare condition. In this case report, a case of focal epilepsy due to the presence of a cavernous angioma in the region of cortical motor area related to tongue movements is described.

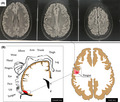

## 1 INTRODUCTION

Involuntary movement of tongue is a clinical manifestation related to neurological conditions affecting central or peripheral nervous system. Potential situations leading to isolated tongue tremor or causing tongue tremor as the first manifestation were described as acute cortical infarction,^1^ Wilson's disease, radiosurgery, brain tumor, electrical injury, chronic alcoholism essential tremor, side effects induced by different drugs,^2^ Parkinson's disease, degenerative disorders of basal ganglia,^3^ and Arnold chiari malformation.^4^ Etiologies leading to focal epilepsy can be a causative factor for tongue tremor as an isolated manifestation or in companion with other symptoms. Epilepsia partialis continua following the conditions such as Rasmussen's and para‐neoplastic limbic encephalitis can be presented with epilepsy‐induced tongue tremors.^5^ Regarding the fact that tongue tremor may be an isolated manifestation of above‐noted conditions, it is of importance for physicians to take appropriate clinical and paraclinical approach to extract the correct diagnosis out of this presentation.

Herein, we present a patient with epileptic tongue tremor as the main manifestation of a cavernous angioma.

## 2 CASE REPORT

A 36‐year‐old right‐handed female was referred to our Movement Disorders Clinic due to episodic involuntary tongue movement for 10 days, each episode lasts approximately 30 to 45 seconds and 5 to 6 times per day. During the attack, she was completely oriented and could talk smoothly and could inform others when the attacks were about to start. She did not mention any difficulties in swallowing. Also, she has bilateral hand tremor since age of 20. The patient also reported a familial history of Parkinson disease in her paternal grandfather. She did not mention any past medical history of head trauma, cerebrovascular, neurological, and neurodegenerative diseases.

During examination, the attack occurred which was a rhythmic movement of tongue, lasted around 20 s without loss of awareness (Video S1).

The fundoscopic examination showed no abnormal finding, including Kayser–Fleischer ring. Somatosensory, pyramidal, and cerebellar examination were unremarkable. She had bilateral action and intention hand tremor, with no sign of rigidity and bradykinesia.

**FIGURE 1:**
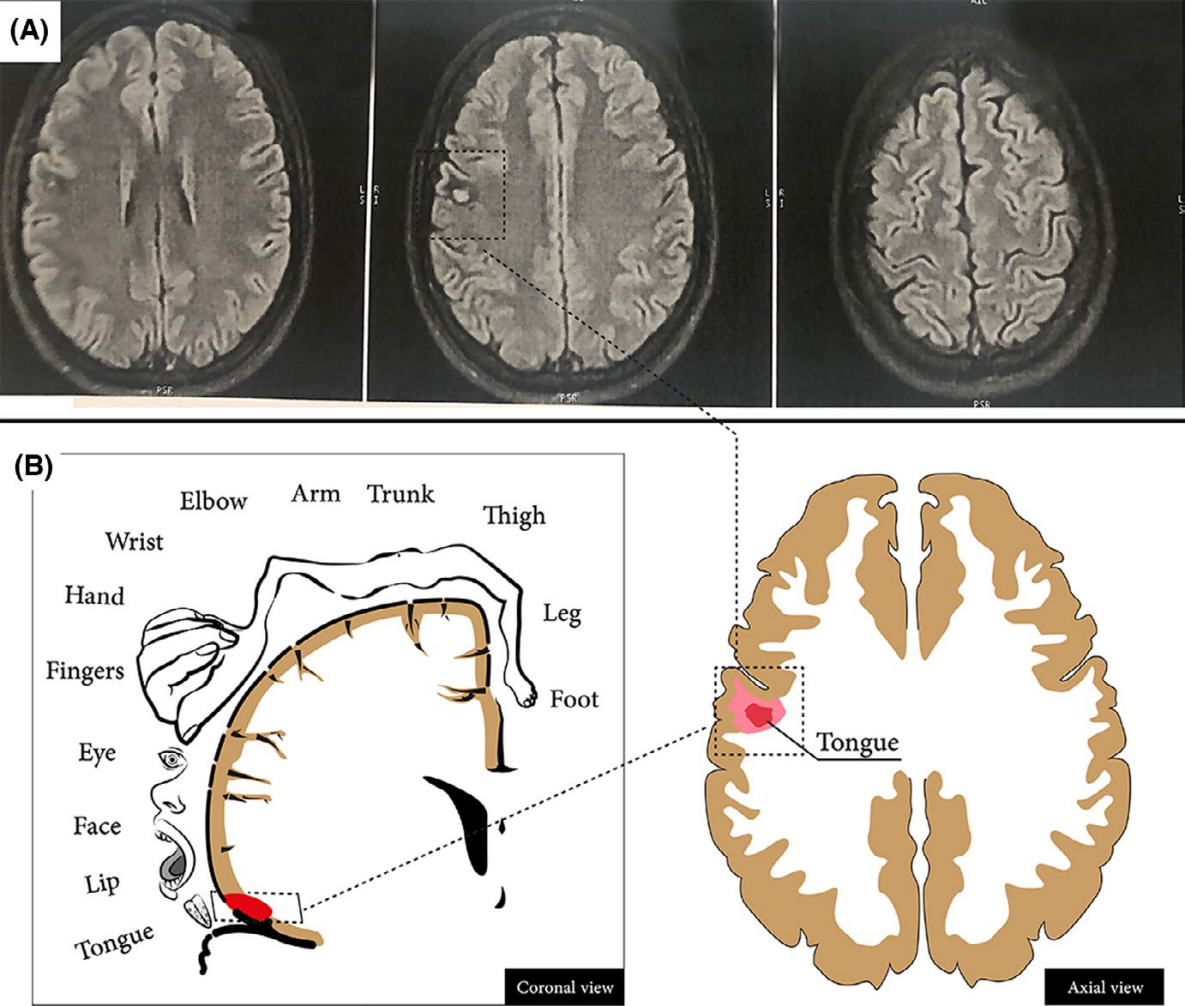
(A) Axial FLAIR imaging demonstrated focal hyperintensity with hypointense suggesting cavernous angioma. (B) Homunculus presentation of the tongue demonstrated the involvement of the tongue motor cortex

Blood samples for thyroid and liver function tests, electrolytes and renal function tests showed no abnormal finding, urine ceruloplasmin and blood copper and were normal. Brain MRI showed the presence of cavernous angioma in the motor area of right cortex (Figure 1). DaTscan showed no abnormality. Interictal EEG was normal.

Lacozomide was started with 100 mg twice a day for her for the diagnosis of seizure and dramatic response observed after 3 days when abnormal movements resolved.

## 3 DISCUSSION

Tongue tremor as the single or the first manifestation is previously described in conditions such as acute cortical infarction,^1^ Wilson's disease, radiosurgery, brain tumor, electrical injury, chronic alcoholism essential tremor, side effects induced by different drugs, such as neuroleptic‐induced tardive dyskinesia,^2^ Parkinson's disease and degenerative disorders of basal ganglia,^3^ and Arnold Chiari malformation.^4^

Focal motor seizures are identified based on their manifestation of epileptic spasms, automatism, clonic, atonic, hyperkinetic, tonic, and myoclonic seizures; in which the responsible neuronal network is localized in one hemisphere, at least in the beginning of the disease. One of the etiologies described for seizures, whether focal of generalized, is structural abnormalities,^6^ in which cavernous angioma can be included.^7^ Focal epilepsy leading to tongue movements is reported in previous studies and case reports. Holtzman and colleagues reported a case of clonic tongue movements in the case of a focal epilepsy due to meningioma which was accompanied by dysphasia and inability to talk, in addition at the onset of the attack, the patient had numbness on the right side of the tongue.^3^ A case of clonic movements of the tongue and unilateral facial drooping impression associated with epileptic seizure was reported previously.^8^ Brainstem pilocytic astrocytoma, associated with right side ataxia and symptoms of raised ICP, was reported in a patient, which the symptoms were replaced with isolated tongue tremor some days after surgical therapy.^9^ Acute cortical ischemia and infarction.

Compared with previous reports of hyperkinetic and other movement disorders of tongue related to focal epilepsy with a structural origin, in which epileptic symptoms involved other parts of body in addition to the tongue, our case report is the first to report focal epilepsy due to a cavernous angioma manifested with isolated tongue tremor as the sole symptom.

## CONFLICT OF INTEREST

There are no conflicts of interest related to this case report.

## AUTHOR CONTRIBUTIONS

MS has made substantial contributions to conception and design and revised and gave final approval of the version to be published. KR was involved in drafting the manuscript and revising it. AM and AB were involved in drafting the manuscript. ME agreed to be accountable for all aspects of the work in ensuring that questions related to the accuracy or integrity of any part of the work are appropriately investigated and resolved.

## ETHICAL APPROVAL

We confirm that we have read the Journal's position on issues involved in the ethical publication and affirm that this work is consistent with those guidelines. The present paper is also approved by the Ethics Committee of the Shahid Beheshti university of medical sciences. The patient has given written and informed consent for online publication of her videos (Ethics code: IR.SBMU.RETECH.REC.1400.559).

## CONSENT

Written informed consent was obtained from the patient for the publication of this case report and any accompanying images and videos. A copy of the written consent is available for review by the Editor‐in‐Chief of this journal.

## Supporting information

Video S1

Video S2

## Data Availability

Data are available on request.
