# Supplementary figures and images for: Focal epilepsy presenting as tongue tremor: A case report
Source: Clin Case Rep. 2022 Feb 23;10(2):e05478. doi: 10.1002/ccr3.5478 (PMC8864572; doi:10.1002/ccr3.5478)

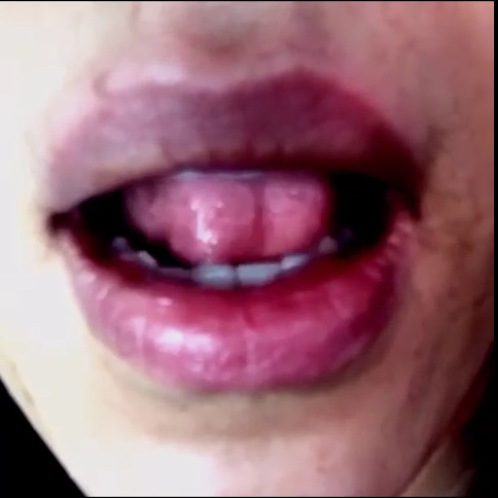

Supplement: Supplementary file 1 — Video S1 [file CCR3-10-e05478-s001.png]
